# Seasonal reversible size changes in the braincase and mass of common shrews are flexibly modified by environmental conditions

**DOI:** 10.1038/s41598-019-38884-1

**Published:** 2019-02-21

**Authors:** Javier Lázaro, Moritz Hertel, Marion Muturi, Dina K. N. Dechmann

**Affiliations:** 1 Max Planck Institute for Ornithology, Department of Migration and Immuno-Ecology, Am Obstberg 1, 78315 Radolfzell, Germany; 2 University of Konstanz, Department of Biology, 78457 Konstanz, Germany; 3 Max Planck Institute for Ornithology, Department of Behavioral Neurobiology, Eberhard-Gwinner-Str., 82319 Seewiesen, Germany

## Abstract

The growth of the vertebrate skull and brain is usually unidirectional and more or less stops when animals are adult. Red-toothed shrews break this rule. They seasonally shrink and regrow brain and skull size by 20% or more, presumably to save energy when conditions are harsh. The size change is anticipatory of environmental change and occurs in all individuals, but it is unknown whether its extent can be modulated by environmental conditions. We kept shrews under different conditions, monitored seasonal changes in skull size with series of X-rays, and compared them with free ranging animals. We found extensive differences in the pattern of skull size change between experimental groups. Skull size of shrews kept at constant temperature showed a steady decline, while the skull size changes of free ranging shrews and captive individuals exposed to natural temperature regimes were identical. In contrast, body mass never reached the spring values of free ranging shrews in either captive regime. The extent of this adaptive seasonal pattern can thus be flexibly adapted to current environmental conditions. Combining reversible size changes with such strong phenotypic plasticity may allow these small, non-hibernating predators with high metabolic rates to continue being successful in today’s changing environments.

## Introduction

Phenotypic plasticity is the capacity of individuals and populations to change their phenotype in response to changes in the environment^1^. Describing phenotypic plasticity can elucidate important questions about the mechanisms of organismal and tissue change at different levels, and also give insight into its role during evolution (e.g. genetic assimilation^1^). Determining the flexibility of a trait across a range of external conditions (i.e. the reaction norm), would allow predicting the adaptive capacity of a population when facing an expected change in the environment, such as climate change^2^.

In many vertebrates there are individual morphological and physiological adaptive transformations between seasons that aid survival during periods of low resources, or increase performance during the breeding season. The cyclic, predictable fluctuations in the environment caused by seasons thus provide a good framework for the study of phenotypic plasticity^3–6^. An outstanding case of seasonal transformation is the morphological change of some species of shrews and mustelids^7–10^. This has been best studied in the common shrew, *Sorex araneus*, a small non-hibernating predator with exceptionally high metabolic rates^11^. Born in early summer, juvenile shrews quickly reach a first growth peak (“summer juveniles”). Then, in anticipation of harsh winter conditions, body size begins to decrease in late summer reaching a minimum size around February (“winter subadults”). Finally, the shrews regrow with the onset of spring and reach a second size peak as they become sexually mature in the second and last summer of their short lives (“second summer adults”). This seasonal size change, called Dehnel’s phenomenon^12,13^, includes a shrinking of the braincase by 15–20% from first summer to winter and a later regrowth by 8–15% in spring along with a profound variation in overall body mass^10,12,14–16^. Simultaneously, brain mass of these shrews decreases by 10–26% followed by a regrowth of 9–16%^16–19^. These individual, seasonal, reversible size changes are thought to be a plastic adaptation to winter conditions^7,9,16,20,21^. A smaller animal also needs to find and ingest fewer resources in the winter, which may mean the difference between death and survival. Disproportionally reducing energetically expensive tissues such as the brain^22,23^ might further decrease shrews’ energy expenditure during the period of low resource availability^11,24–26^. However, bone and brain size are not usually flexible traits, thus this phenomenon is an unusual and remarkable case of phenotypic plasticity.

The size change occurs in every individual and in anticipation of harsh conditions. Unlike the similar size change in marine iguanas^27^ it is thus clearly not a reaction to exceptional harsh conditions, but a regular seasonal pattern^9,10,16^. Previous studies of *S. araneus* described  greater intensity of the skull size changes in Northern and Eastern populations compared to Central Europe^7^. This, supports that the severity of winters may be a driver of the evolution of this phenomenon. In contrast, there is no latitudinal difference in the changes in body mass^28^. However, body mass, which commonly changes seasonally in many animals, does not follow the same pattern as the skull, brain and other organs in the shrews and may not be a good measure for this phenomenon^10,15^. Similarly, the environmental cues that trigger the shrinkage/regrowth process are not yet understood. For example, skull height changes of captive shrews under different day length regimes were not affected by photoperiod^29^. Importantly, previous studies used measurements from different individuals during each season instead of time series of the same individual. Therefore, it remained unclear whether this phenomenon is fixed in its pattern or can be modulated at the individual level. Only detailed comparative studies that follow individual responses under different environmental conditions will help us tease apart the baseline seasonal pattern from modulations caused by current environmental conditions. The common shrew offers a unique opportunity to link the ontogeny of skull and brain to external triggers, which will ultimately reveal the evolutionary drivers that determine skull and brain size in a mammal.

In a recent study we documented the individual nature of the seasonal changes in skull size of recaptured wild shrews using repeated X-rays^10^. We now compared these data with changes in skull size and body mass of captive shrews kept under different conditions using the same method. Building on the evidence for latitudinal differences in the extent of the phenomenon between populations, we hypothesized that even though Dehnel’s phenomenon is a regular seasonal pattern in this species, the intensity of the changes in skull size and body mass can be modulated by current environmental conditions. We tested our predictions by comparing four groups where we manipulated temperature and food availability. The first group was kept in captivity with *ad libitum* diet, natural daylight cycles, but constant temperature (“constant temperature” experimental group). If temperature is a trigger or modulator of Dehnel’s phenomenon we expected the pattern to be less pronounced in these individuals. The second group was kept under natural daylight cycles, and the same food regime as the first group, but at the temperatures that wild conspecifics would experience (“natural temperature”). This setup allowed us to test for the effect of food availability on Dehnel’s phenomenon. We expected the pattern in these individuals to be less pronounced than in the free-ranging animals, which depended on food they foraged for themselves. Both, “constant temperature” and “natural temperature” consisted of individuals captured as juveniles in summer during the first size peak. A third group consisted of individuals that were kept under the same conditions as “natural temperature”, but were caught later, during the winter size minimum (“natural late”). This allowed us to test if the age at which wild individuals are brought in captivity can affect Dehnel’s phenomenon. We then compared changes in skull size and body mass of these three experimental groups with the previously collected data from recaptured free ranging individuals (“free-ranging”^10^). Our results show the astounding flexibility of the seasonal changes of individual skull size and body mass of common shrews.

## Results

### Skull measures

**Figure 1:**
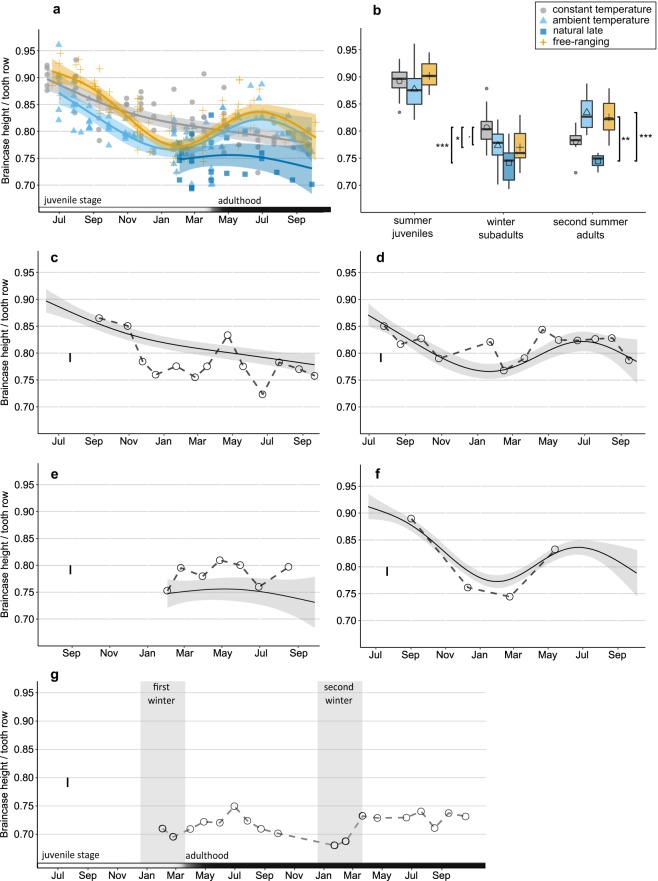
(**a**) Variation in BCH over time in the four groups. Lines and shadowed areas represent fitted GAMMs and 95% confident intervals respectively. (**b**) Means (symbols), medians and quantiles (boxes) for each group in the three seasons. Vertical segments indicate significant differences from the posthoc comparisons between groups in each season, as revealed by the LMM. Only comparisons within seasons are shown here. ^·^P = 0.06; *P < 0.05; **P < 0.01; ***P < 0.001. Examples of short-term changes in BCH of four individuals from (**c**) “constant temperature”, (**d**) “natural temperature”, (**e**) “natural late” and (**f**) “free-ranging”. Dots connected with dashed lines indicate repeated measurements of BCH for each individual. Solid lines and shadowed areas represent fitted GAMMs and 95% confident intervals respectively, each individual’s panels includes the curve corresponding to all animals from its group. (**g**) Repeated measurements of BCH in a long lived individual illustrating a second shrink-regrowth cycle. Vertical black lines at the left of each panel (c–g) represent the technical error of measurement as calculated in Lázaro *et al*.^10^.

Our data for standardized brain case height (BCH, Fig. 1a) revealed changes across the seasons in significant non-linear patterns in all four groups (GAMM (Generalized Additive Mixed-effects Model), n = 271, e.d.f. (“constant temperature”) = 1.861, e.d.f. (“natural temperature”) = 3.605, e.d.f. (“natural late”) = 3.103, e.d.f. (“free-ranging”) = 3.889, P (smooth term) < 0.001, adjusted r-squared = 0.54). The curve for animals where we modified only one parameter (i.e. *ad libitum* food, but natural temperature and day length “natural temperature”) followed the same seasonal decrease-regrowth patterns as those from the wild recapture study (“free-ranging”; Fig. 1a). In contrast, measurements from individuals where two parameters were modified (*ad libitum* food and constant temperature, but natural day length, i.e. “constant temperature”), showed a continuous decline in BCH over time, but not the expected reversible seasonal pattern. Finally and confusingly, those animals (“natural late”) that were also exposed to the same single modified parameter as the “natural temperature” ones (i.e. *ad libitum* food, but natural temperature and day length), but which we only took into captivity during winter when their BCH was lowest, did not show the regrowth observed in “free-ranging” and “natural temperature”, but a parabolic curve instead.

The comparison between LMMs (Linear Mixed-effects Models) for BCH revealed M1 (AICc = −455.5) as the best fitted model, which significantly differed from both M2 (AICc = −435.9) and M3 (AICc = −432.0, Anova P < 0.001). M1 indicated a significant effect of season and group as well as their interaction. We therefore proceeded to compare the differences in BCH between all groups between and within seasons with a Tukey test. For P values from pairwise comparisons see Table 1.

**Table 1:** P-values derived from Tukey pair-wise comparisons between seasons of each treatment group and between treatments of the same season.

Treatment	Season	Constant temperature			Natural temperature			Natural late		Wild			
		juv	sub	ad	juv	sub	ad	sub	ad	juv	sub	ad	
constant temperature	juv		***	***	0.91					0.37–			BCH
	sub	0.12		0.16		0.06		***			**		
	ad	*	0.74				0.12		0.25			0.37	
natural temperature	juv	**				***	**			0.10			
	sub		***		***		***	0.08			0.86		
	ad			0.85	0.09	***			**			0.91	
natural late	sub		0.29			0.07			0.78		0.23		
	ad			0.74			0.98	*				*	
free-ranging	juv	0.47			0.19						***	***	
	sub		***			0.62		0.39		***		***	
	ad			***			***		***	***	***		
		body mass											

The upper part of the table depicts the results from BCH; the lower part shows the results from body mass. “juv” = summer juveniles; “sub” = winter subadults; “ad” = second summer adults. When statistically significant, P-values were symbolized by “*” < 0.05, “**” < 0.01 and “***” < 0.001.

**Figure 2:**
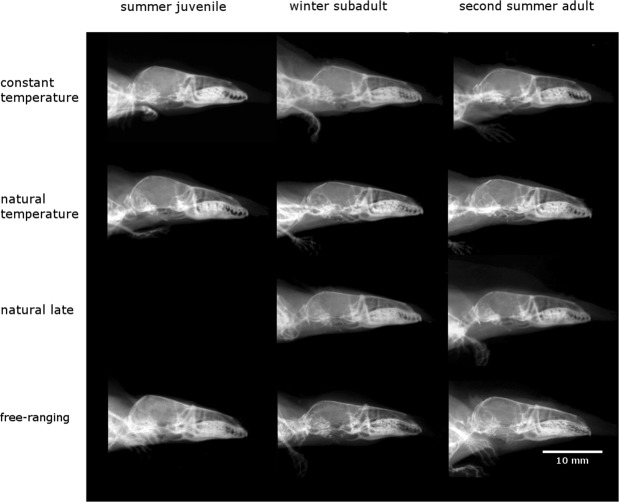
X-ray images of shrew skulls from the four groups. Each row depicts images of the same individual along the three stages (two stages for “natural late”). The images of the “free-ranging” individual are adapted from Lázaro *et al*.^10^.

Shrews kept under “constant temperature” significantly declined in BCH from summer juveniles to winter subadults, but this decline was less intense than the decline experienced by the “free-ranging” shrews which were exposed to natural conditions, leading to a difference between the subadults of these two groups (Fig. 2, Table 1). Also, “constant temperature” shrews did not regrow their BCH from winter to their adult stage in the following spring, but continued to decline. BCH of both “natural temperature” shrews where only food availability was modified and “free-ranging” shrews recaptured from the wild, decreased significantly from juveniles to subadults and then regrew from subadults to adults (Figs 1b, 2, Tables 1 and 2). We found no differences between these two groups at any of the main life/size stages (Table 1). Thus, captive shrews kept under natural daylight and temperature, but with artificial *ad libitum* food showed the same seasonal pattern as “free-ranging” animals. Interestingly, we did not observe the regrowth from subadults to adults in the “natural late”. They remained smaller than both “natural temperature” (P < 0.01) and “free-ranging” although just like in the “natural temperature” shrews, only food availability was modified once they were captured (Fig. 2, Table 1).

**Table 2:** Corrected braincase height of the shrews in the four groups at the three age/size stages.

Season	Summer Juveniles			Winter Subadults			Second Summer Adults			Dif. Juv.-sub.(%)	Dif. Sub.-ad.(%)
treatment	n	mean ± SE	SD	n	mean ± SE	SD	n	mean ± SE	SD		
constant temperature	12	0.892 ± 0.008	0.029	11	0.807 ± 0.011	0.036	8	0.780 ± 0.010	0.027	−9.6	−3.3
natural temperature	11	0.877 ± 0.012	0.039	17	0.773 ± 0.007	0.031	10	0.834 ± 0.011	0.034	−11.8	7.9
natural late	—	—	—	9	0.742 ± 0.012	0.036	3	0.744 ± 0.011	0.019	—	0.4
free-ranging	9	0.902 ± 0.009	0.026	17	0.770 ± 0.007	0.029	8	0.825 ± 0.013	0.036	−14.6	7.1

Our data also revealed some variation in skull length (SKL) and braincase width (BCW) across time. However, the seasonal changes in the patterns, although similar, were less profound than those in BCH, and similar to what was found in previous studies. For details on SKL and BCW see the Supplementary Information (Figs S1 and S2).

Obtaining monthly measurements in captive shrews provided relatively high time resolution data. In contrast, in the mark-recapture study with the free ranging individuals, animals were captured much less often and irregularly (although trapping efforts were also made monthly). We found small short-term fluctuations in BCH of captive individuals (Fig. 1c–e). Such short-term directional changes may or may not occur in free-ranging shrews, but were not detectable at the temporal resolution at which we were able to catch them (Fig. 1f).

One captive individual from the “natural late” shrews, captured during winter as small-brained subadults, passed a second winter and survived until October of its third calendar year. This allowed us to collect exceptional albeit anecdotal data from this long-lived individual, as shrews rarely pass a second autumn in the wild. The X-ray images of this individual’s BCH revealed a complete decrease-regrowth cycle during the second year (Fig. 1g). BCH increased 7.8% from February to June; then it decreased 9.2% from June to January and finally regrew 8.9% until the shrew’s death in July.

### Body mass

**Figure 3:**
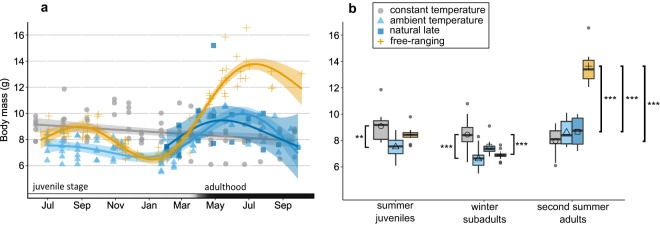
Seasonal variation of body mass in the four groups of shrews with (**a**) fitted GAMMs, and (**b**) means, medians and quantiles for each group, indicating significant differences from pairwise posthoc comparisons. Symbols and colours are the same as in Fig. 1a,b.

Body mass also followed a seasonal pattern of decrease and regrowth (Fig. 3a), but here all experimental groups (“constant temperature”, “natural temperature” and “natural late”) differed from the “free-ranging” one. Even “natural temperature” shrews, which showed the same skull regrowth pattern, differed from the “free-ranging” ones in mass during the spring increase. While mass of second summer adults in the free-ranging shrews was 61.2% higher than during their first summer, second summer mass was only 14.8% higher than during their first summer in “natural temperature” (Fig. 3a; Table 3). This is surprising as “natural temperature” shrews differed from free-ranging ones only in that they received *ad libitum* food. In contrast, the seasonal mass pattern was not significant in the “constant temperature” shrews. The curve in body mass changes was most pronounced in “free-ranging” shrews, both during the decrease and the regrowth (Fig. 3a; Table 3). Mass gain patterns of “natural temperature” and “natural late” were similar in shape, but weaker (GAMM, n = 275; e.d.f. (“constant temperature”) = 1.000; e.d.f. (“natural temperature”) = 3.877; e.d.f. (“natural late”) = 2.615; e.d.f. (“free-ranging”) = 3.972; P(smooth term for “free-ranging” and “natural temperature”) < 0.001; P(smooth term for “constant temperature”) > 0.05; P(smooth term for “ambient late”) < 0.05; adjusted r-squared = 0.53).

**Table 3:** Body mass (g) of the shrews in the four groups at the three age stages.

Season	Summer Juveniles			Winter Subadults			Second Summer Adults			Dif. Juv.-Sub.(%)	Dif. Sub.-Ad.(%)
treatment	n	mean ± SE	SD	n	mean ± SE	SD	n	mean ± SE	SD		
constant temperature	13	9.09 ± 0.30	1.10	12	8.45 ± 0.36	1.26	9	7.96 ± 0.38	1.13	−7.0	−6.2
natural temperature	12	7.51 ± 0.25	0.86	18	6.61 ± 0.17	0.75	8	8.62 ± 0.37	1.06	−12.0	23.3
natural late	—	—	—	8	7.54 ± 0.25	0.71	4	8.67 ± 0.67	1.34	—	13.0
free-ranging	9	8.46 ± 0.21	0.64	14	6.90 ± 0.09	0.35	8	13.64 ± 0.49	1.39	−18.4	97.7

LMM comparisons for body mass revealed a best model M1 (AICc = 316.34), which significantly differed from M2 (AICc = 431.38, Anova P < 0.001) and from M3 (AICc = 440.38, Anova P < 0.001). This indicated a significant effect of season and group at both factor and interaction levels.

As already suggested by the GAMM, shrews kept at “constant temperature” did not change their body mass significantly, neither from juveniles to subadults, nor from subadults to adults (Fig. 3b, Table 1). “Natural temperature” and “free-ranging” declined in body mass from juvenile to subadult (P < 0.001). This was followed by a regain of mass from subadults to adults. The shrews kept at natural conditions with *ad libitum* food that were captured only as subadults (“natural late”), which had shown no spring regrowth of the skull, showed a mass increase here. However, the regrowth of body mass in all experimental groups was less pronounced than in the free-ranging shrews, leading to a significant difference at the adult stage (Fig. 3b, Table 1).

## Discussion

Our results indicate that seasonal plasticity in shrews’ skull size and body mass can be strongly modified by external factors, even though the changes are anticipatory of a change in conditions and naturally occur in all individuals. The differences that we observed between our experimental groups clearly support a link between the morphological changes and environmental variables. They also emphasize the flexibility of this phenomenon. Captive shrews kept at constant temperature (“constant temperature”) steadily decreased in size and did not regrow, completely breaking the curved pattern observed in shrews exposed to natural temperature (“natural temperature”, “free-ranging” and, to a lesser extent, “natural late”). Ambient temperature thus clearly had an effect on the process, even though the lack of a significant regrowth in the animals caught during the minimum size stage (“natural late”) cannot be explained by temperature. However, even at constant temperature shrews exhibited a size change, i.e. a steady decrease in BCH. Such a pattern is rarely observed in vertebrates and then usually in response to harsh conditions, which these *ad libitum* fed animals did not experience^27,30^. Clearly, there are additional intrinsic or external triggers involved in the change in skull size.

Also, the temperature at which we kept “constant temperature” animals (18 °C) was randomly selected as a rough mean of what the shrews experience in the wild. Triggers inducing a change in the pattern such as switching to the regrowth, could also be the interplay between external variables (for example a certain temperature coinciding with a certain day length). Thus, this relationship and especially also a potential role of photoperiod - an important trigger for seasonal changes in other species - remains far from resolved. That both temperature and photoperiod play a role here is likely, as they are more stable over time and therefore more reliable as seasonal cues than food supply. In fact, some animals respond to photoperiod with changes in bone growth^31–33^. As our experimental groups were all exposed to the same – natural – photoperiod, we cannot draw conclusions about the role of photoperiod based on our results. The only study to date that assessed this variable did not find any effect of photoperiod on Dehnel’s phenomenon^29^. However, as our results show, captive conditions can play a large role here, potentially also masking effects and more studies using longitudinal data will be necessary to disentangle the role of photoperiod from that of other environmental cues.

One important parameter we modified in captive animals was diet: while the free-ranging shrews consumed their natural diet, all of our captives received the same *ad libitum* food mixture. We found no effect of this change in diet on the seasonal patterns of morphological change, as the size change patterns of “free-ranging” and “natural temperature” animals (natural daylight and temperature) were identical. This indicates that the observed winter decrease in skull size and body mass was not a reaction to the current resource landscape, but may be the result of a more long-term evolutionary adaptation^24^. Therefore, although it does not seem to be a trigger, resource availability and/or quality may still have been an important evolutionary driver of the phenomenon.

The short-term direction changes in the size change from month to month in some of the captive individuals emphasizes our lack of understanding of the triggers and modifiers of this seasonal phenomenon (Fig. 1c–g). At the same time, these short-term fluctuations in BCH emphasize the incredible flexibility of this process at the individual level. We can only speculate whether similar patterns underlie the more general curve of size change also in the wild. However we would need much higher recapture rates than the ones achieved with great effort in our mark-recapture study^10^ to document this. Most studies in the past have focused on the three size extremes, summer juveniles, winter subadults, and second summer adults. Our data demonstrate the importance of increasing the temporal resolution, as well as the necessity for more carefully designed captive studies.

Importantly, the repeated seasonal pattern we observed in the example of an exceptionally long-lived female, which showed a second cycle of the pattern, demonstrates that the phenomenon is not purely an ontogenetic process (Fig. 1g). The short natural life-span of wild common shrews usually prevents similar observations although a beginning of a second decrease in late summer adults can sometimes be observed^7,10,12,16^.

We designed our experiments based on the evidence for a geographical pattern in Dehnel’s phenomenon. Seasonal differences in skull size are more pronounced in northern and eastern populations in Europe^7^, where winter conditions are harsher. Our results indicate that in addition to these large-scale regional patterns, the short-term intensity of the size changes can also be modified by environmental cues and therefore the severity of seasons. Over time, such differences may then have become fixed or partially fixed in different populations by means of genetic assimilation^1^. Similarly, we would also expect an altitudinal gradient, but so far no studies have addressed this aspect. Finally, following this line of thought, one may also predict a severe effect of the currently rising global temperatures on animals exhibiting this phenomenon. In areas where winters are becoming milder^34^, the intensity of the seasonal size changes may diminish or disappear depending on the effects on prey and other aspects of community structure. This is supported by the fact that in a southernmost Spanish population of a closely related species, *S. coronatus*, no evidence for Dehnel’s phenomenon was found^35^.

Our results clearly show a large reaction norm of this phenomenon to environmental factors. Temperature and possibly also other environmental cues play an important role. However, our results from shrews kept at constant temperature and those captured later in the size change cycle show that this is not nearly the entire story. Some differences between groups might be at least partially due to physiological stress-induced reactions^36,37^. Shrews have exceptionally high metabolic rates and are sensitive to stress. Captive conditions are likely to be extremely important. In a previous study, for example, captive shrews showed less pronounced seasonal changes than wild ones^29^. Others had problems with shrews becoming obese in captivity^29,38^, evidence of reduced activity and or energy expenditure, which may also have had effects on the expression of Dehnel’s phenomenon. We added a running wheel, which the animals use extensively, as well as a more structured and large home cage (Fig. S3). This seems to have alleviated these effects in our study.

However, mimicking natural conditions is difficult. For example, the light regime our indoor captives (“constant temperature”) were exposed to, was as natural as possible. We used daylight-spectrum lamps and relatively dim settings to mimic the environment of these litter dwelling animals. Nonetheless, we cannot exclude a potential lab artifact of light influencing the pattern of decrease we observed in this treatment. Similarly, we do not entirely understand the faint, almost absent regrowth of BCH in the “natural late” animals, which we caught during the small subadult winter stage. Here, in particular, greater stress sensitivity of this specific age/seasonal stage may have played a role. Thus, we want to use our results to emphasize the great flexibility of the size change at the individual level rather than weighing the influence of various environmental variables on Dehnel’s phenomenon against each other in too much detail. More targeted research, incorporating hormones and other physiological processes will be necessary to tease apart the evolutionary drivers from the triggers of this phenomenon.

Our results from body mass also indicate that we are far from completely understanding the triggers and modifiers of Dehnel’s phenomenon. While the modeled results from the BCH of “free-ranging” shrews and those kept at natural daylight and temperatures were identical, there were large differences between the body mass curves of these two groups. The “natural temperature” animals did not show the typical profound weight gain along with the skull regrowth in spring. This indicates that there was an influence of captivity even in the *ad libitum*-fed “natural temperature” group. We suspect that the regrowth in spring happens in preparation for the terminal reproductive period. By preventing the shrews, which were housed in individual cages, from meeting potential mating partners in the spring we may have removed a potential trigger for body mass growth. Mass has been frequently used as a proxy for Dehnel’s phenomenon^28,39,40^, and some mass change is to be expected as not only the brain, but also several other organs exhibit strong size changes between seasons^15^. However, many species show reversible mass changes as a result of fat storage and use in the course of winter. Thus, how strongly mass changes in other organs and tissues are a fundamental part of Dehnel’s phenomenon also remains unresolved.

Our main response variable, BCH, is strongly correlated with brain mass^16^. During the seasonal shrinking and regrowth of brain size, there are also profound changes in brain structure^19,21,41^ with consequences for cognitive performance^16^. This demonstrates that though probably adaptive as an energy saving strategy, the seasonal loss in brain size incurs a compromise. The extreme plasticity of the skull and correlated brain size and structure, may force the shrew to optimize the trade-off between responding to energetic limitation and maximizing cognitive performance. The high level of individual flexibility we observed may contribute to this. A better understanding of this process, and of its triggers, causes, and mechanisms, will help us understand the forces that shape the ontogeny and evolution of the skull and brain. Finally, researching the genetic, cellular and molecular processes beneath the seasonal but flexible changes in bone and brain might lead to promising medical applications related to tissue regeneration.

## Methods

### Trapping and recapturing specimens

All fieldwork took place in the surroundings of our Institute in Möggingen, Germany (47°46′04.70″N, 8°59′47.11″E). We trapped common shrews (*Sorex araneus*) with wooden live-traps baited with mealworms and checked them at two-hour intervals (PPUH A. Marcinkiewicz, Rajgród, Poland). We determined shrews’ age (first summer juveniles, winter subadults or second spring/summer adults) mainly based on the season of capture. To distinguish between juveniles and adults during the brief period of overlap, we checked for the degree of development of the testes and mammary glands, body mass, and fur appearance^42,43^. We assigned shrews to four groups: “constant temperature” (N = 12 individuals); “natural temperature” (N = 14); “natural late” (natural temperature, trapped in winter, N = 5); and “free-ranging” (n = 37). Data from “free-ranging” animals were collected during a previous mark-recapture study^10^, from June 2014 to October 2015 with monthly trapping/recapture efforts. At each recapture, we took the shrews’ body mass and obtained an X-ray image of the skull for measuring (see details below). We kept shrews, assigned to the other groups, in captivity after first capture, and weighed and X-rayed them monthly until they died. We individually marked all shrews at first capture with subcutaneous passive integrated transponders (UNO PICO, 7 × 1.5 mm, UNO BV, Zevenaar, Netherlands). We trapped shrews for “constant temperature” from August 2014 to January 2015 and for “natural temperature” between June and December 2015 and 2016, i.e. during the shrinking phase of the phenomenon. For the last group, “natural late”, we trapped new animals in January and February 2016, i.e. during the expected minimum size in winter.

### Captive conditions

**Figure 4:**
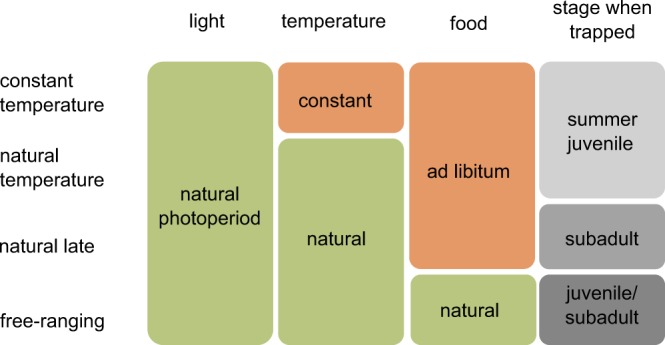
Illustration of the experimental design showing the configuration of external factors in the four experimental groups.

We kept animals assigned to each of the three captive groups (“constant temperature”, “natural temperature” and “natural late”) in double-cage systems. One cage (38 × 25 × 30 cm) contained a thick layer of natural soil, and a second layer of hay in which a plastic tube and a terracotta flowerpot were buried for nesting. This cage was connected to a second one via a plastic tube and contained a thin layer of soil, a running wheel, food and water dishes, and another inverted terracotta pot with hay for bedding (Fig. S3). We fed all captives daily *ad libitum* with a special food mixture^44^. In addition, they received 2 g of mealworms (larvae of *Tenebrio molitor*) and 2 earthworms (*Lumbricus terrestris)*. Water was provided *ad libitum* (Fig. 4).

“Constant temperature” shrews were kept in a room under controlled ambient conditions. The room was illuminated with artificial light (using daylight spectrum bulbs) that followed the local natural light/dark regime. The room temperature was kept constant (18 °C) throughout the experiment. Outdoors captives (“natural temperature” and “natural late”) were kept in an outside aviary, protected with a roof and exposed to natural temperature and daylight (Fig. 4).

### X-ray images

We X-rayed the skull at each capture for “free-ranging” shrews and once a month for captives. For the X-ray images, we anesthetized shrews in an induction chamber (oxygen flow rate 1 l/min, 5% Isoflurane) with a Titus anaesthesia system (Dräger, Lübeck, Germany). Once anesthetized, we placed animals into a form-fitting foam bed that ensured standardized skull position for all individuals. We then placed the foam bed into a Faxitron MX 20 cabinet (26 kV, 6 sec) and took ventral and lateral X-ray images of the skull. We used an OPG Imaging Plate (Gendex) and a scanner (DenOptix/Gendex) to develop images. For details see also Lázaro *et al*.^10^.

### Skull measurements

We measured skull dimensions on the digital X-ray files using the software Image-J^45^. We measured braincase width (BCW) as the greatest lateral diameter of the braincase; skull length (SKL), from the anterior most projection of I1 to the occipital condyle; and braincase height (BCH), from the dorsal outline of the braincase to the orthogonal line defined by the ventral outlines of the pterygoid process and occipital condyle, passing over a constant proportional distance (k) on the line defined by SKL^10^ (Fig. S4). We divided these three measurements by the maxillary tooth row length, which remains constant across seasons, for standardization^10^. All measurements were taken blind by a single observer (JL) to avoid any possible biases.

### Statistical analyses

To assess the seasonal variation in skull dimensions and body mass in all groups, we first tested for the expected non-linear patterns using Generalized Additive Mixed-effects Models (GAMMs)^46^ for our four variables of interest (BCH, SKL, BCW and body mass). For these GAMMs we used an age-corrected Julian day of the year (jday) as a non-parametric term to quantify potential nonlinear patterns. To calculate jday for juveniles (trapped in their year 1), we set 1^st^ of June of year 1 to jday = 1 until 31^st^ of May of the following year (jday = 365). For adults (trapped in their year 2) jday is 365 + jday of year 2. We included group as a parametric term and individual as a random effect. We added a smoothing function to jday which included five knots in a thin regression spline and we used a Gaussian distribution. This approach allowed us to test for significance of nonlinear patterns in each group. We checked for temporal autocorrelation and partial autocorrelation in the models using the functions acf and pacf in R. GAMMs were performed with the R package mgcv^47^.

In addition to describing the continuous curves, we also assessed the differences between groups in each of the three main stages of the phenotypic cycle: juveniles in June and July of their first summer; size decreased subadults in January and February; and regrown adults in June and July of their second summer. To analyse patterns of each of our four response variables (BCH, SKL, BCW and body mass) we used four sets of Linear Mixed-effects Models (LMM) with Gaussian distribution and fitted with the maximum likelihood criterion. For each response variable, we ran three LMMs and compared them using Anova. In the first model (M1) we included size/age, stage and group as well as their interactions as fixed effects, and individual as random effect. In the second model (M2) we removed the interaction, so we treated stage and group only as factors. In the third model (M3) we removed group, leaving season as the only fixed effect. We chose the best fit model for each response variable based on the corrected Akaike Information Criterion (AICc). Next, we did pairwise comparisons between groups based on the chosen model. As the different models include different numbers of factors and interactions, the choice of one model lead to a different possible set of pairwise comparisons between all the factors’ levels. Thus, once we had chosen the best model, we performed pairwise comparisons between groups using Tukey tests for multiple comparisons. LMMs were fitted with the R package lme4^48^. Tukey tests were performed with the emmeans R package^49^. All analyses were performed in R 3.5.0^50^.

### Ethical statement

All methods were performed in strict accordance with the protocols for trapping and handling animals, as well as the guidelines for captivity conditions, which were all approved by the Regierungspräsidium Freiburg, Baden-Württemberg (Permit Numbers 35-9185.81/G-11/21, 35-9185.81/G-14/28 and G-15/128).

## Supplementary information

Supplementary Information

Dataset 1

## Data Availability

The dataset supporting this article has been uploaded as part of the Supplementary Material. Additionally, the data on free ranging shrews is available in the Dryad repository: 10.5061/dryad.15r51.
