# Supplementary material for: Seasonal reversible size changes in the braincase and mass of common shrews are flexibly modified by environmental conditions
Source: Sci Rep. 2019 Feb 21;9:2489. doi: 10.1038/s41598-019-38884-1 (PMC6385354; doi:10.1038/s41598-019-38884-1)
Supplement: Supplementary file 1 — Supplementary Information [file 41598_2019_38884_MOESM1_ESM.pdf]

Supplementary Information for:

Seasonal reversible size changes in the braincase and mass of common shrews are flexibly modified by environmental conditions

Authors:

Lázaro, Javier*
Affiliation: Max Planck Institute for Ornithology. Am Obstberg 1, 78315, Radolfzell, Germany
University of Konstanz, Department of Biology, 78457 Konstanz, Germany
Mail: jlazaro@ron.mpg.de

Hertel, Moritz
Affiliation: Max Planck Institute for Ornithology. Eberhard-Gwinner-Str., 82319, Seewiesen, Germany
Mail: hertel@orn.mpg.de

Muturi, Marion
Affiliation: Max Planck Institute for Ornithology. Am Obstberg 1, 78315, Radolfzell, Germany
University of Konstanz, Department of Biology, 78457 Konstanz, Germany
Mail: muturi@orn.mpg.de

Dechmann, Dina K N
Affiliation: Max Planck Institute for Ornithology. Am Obstberg 1, 78315, Radolfzell, Germany
University of Konstanz, Department of Biology, 78457 Konstanz, Germany
Mail: ddechmann@orn.mpg.de

*corresponding author

Results on SKL and BCW

Skull length

The GAMM revealed a significant change across seasons in SKL in all groups except in "late captures". (Fig. S1, GAMM, n = 271, e.d.f. ("constant temperature") = 1.000, e.d.f. ("ambient temperature") = 3.327, e.d.f. ("late captures") = 1.000, e.d.f. ("free-ranging") = 3.698, P (smooth term for "constant temperature", "ambient temperature" and "free-ranging") < 0.001, P (smooth term for "late captures") > 0.5, adjusted r-squared = 0.16). The "constant temperature" group showed a steady decline in SKL, with no spring regrowth. "Ambient temperature" and "free-ranging" shrews underwent a faint shrinking and regrowth change in SKL.

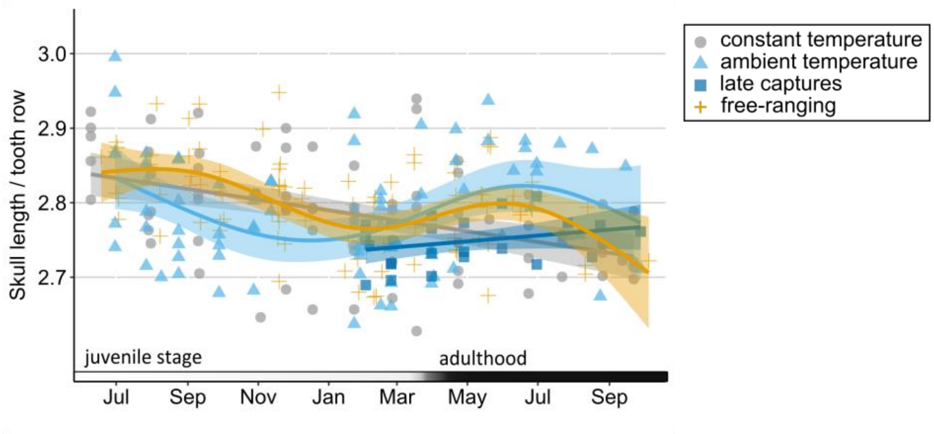

Figure S1. Variation in SKL over time in the four groups. Lines and shadowed areas represent fitted GAMMs and 95% confident intervals respectively.

Our analyses of SKL based on LMMs indicated no significant differences between M1 (AICc = -340.7) and M2 (AICc = -339.91, Anova P > 0.5) and between M1 and M3 (AICc = -345.31, Anova P > 0.5). We found a significant difference between M2 and M3 (Anova, P < 0.001). As M3 showed the lowest AICc value and is the simplest model – only including season as explanatory variable – we concluded that group did not have a significant effect on SKL neither at the factor nor at the interaction level.

Brain case width

Constant and ambient temperature groups showed a smooth non-linear change in BCW and free-ranging animals a slight linear decline. We found no significant change in BCW in "late captures" (Fig. S2 GAMM, n = 253, e.d.f. ("constant temperature") = 2.482, e.d.f. ("ambient

temperature") = 2.213, e.d.f. ("late captures") = 2.297, e.d.f. ("free ranging") = 1.000, P (smooth term for "constant temperature" and "free-ranging") < 0.001, P (smooth term for "ambient temperature") < 0.05, P (smooth term for "late captures") > 0.5, adjusted r-squared = 0.16).

For BCW, we did not find significant differences between M1 (AICc = -398.26) and M2 (AICc = -400.75, anova P > 0.05) and between M2 and M3 (AICc = -401.61, anova P > 0.1). We found a difference between M1 and M3 (anova, P <0.05). Similarly to SKL, we concluded that there is a significant effect of season on BCW, but not of group.

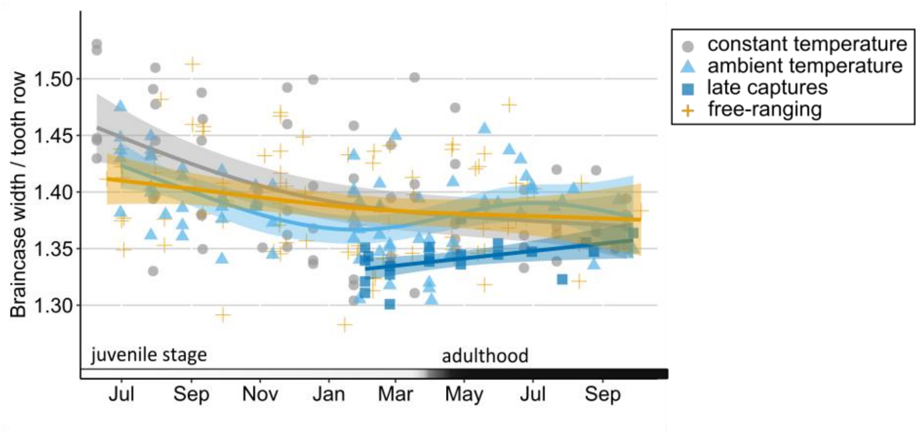

Figure S2. Variation in BCW over time in the four groups. Lines and shadowed areas represent fitted GAMMs and 95% confident intervals respectively.

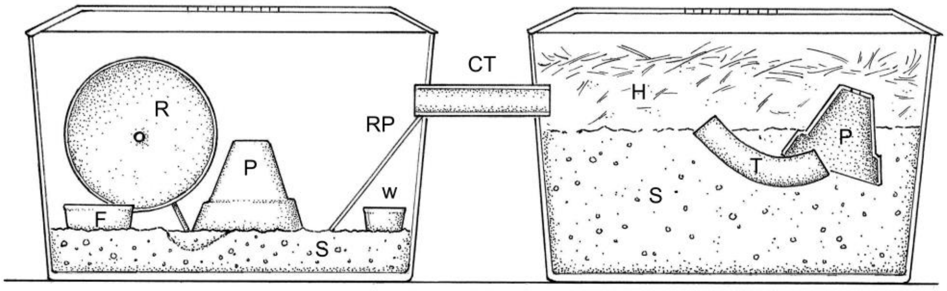

Figure S3. Double cage system for captive shrews. R: running wheel; P: inverted pots for nesting; F: container for food; W: container for water; RP: ramp; CT: connecting tube between cages; S: soil; H: hay; T: tube.

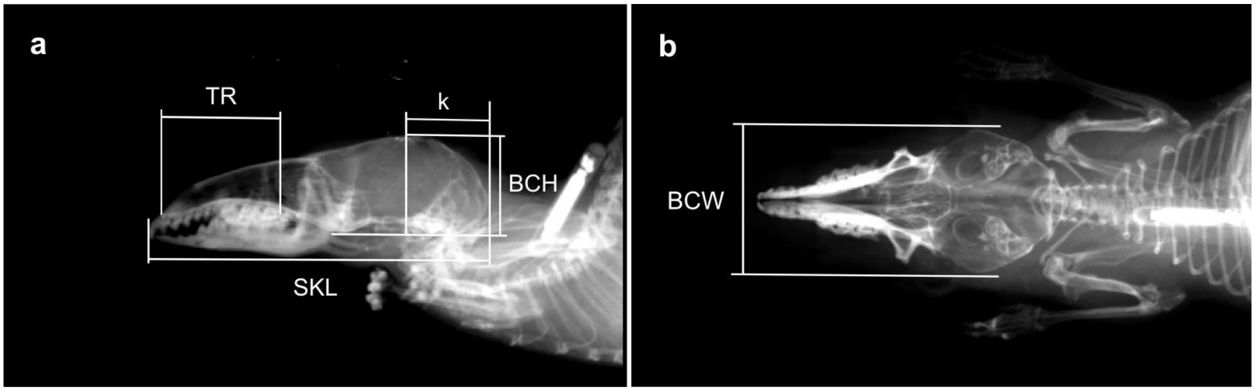

Figure S4. Linear measurements taken from X-ray images of skulls from (**a**) lateral and (**b**) ventral views: braincase height (BCH), skull length (SKL), tooth row length (TR) and braincase width (BCW).
